# Notes and News

**Published:** 1897-01-09

**Authors:** 


### NOTES AND NEWS.

(COLLECTED AND COMMUNICATED.)

THE memorial statue to Surgeon-Major Parke at Dublin has been unveiled by Lord Roberts.

MEASURES are being taken in Italy to prevent the introduction of the plague from Indian ports.

A LEGACY of £200 has been bequeathed to the Middlesex Hospital by the late Mr. William Dorrell.

THE plague in Bombay shows no diminution, and has advanced into the suburbs. The health of European residents is, however, reported as satisfactory.

AMONGST the commemoration events announced for the present year is a fête and demonstration, to be given under the auspices of the St. John Ambulance Association, at the Crystal Palace.

A NEW fever hospital has been erected for Stirling County. It was opened on December 22nd by Mr. G. Ure. The buildings are arranged in four blocks, and the cost has been £7,500.

ONE hundred guineas have been presented to University College Hospital by the members of the West London Dramatic Society, being the proceeds of an entertainment in St. George's Hall.

THE opening of the new operating theatres and chloroform rooms at St. George's Hospital formed an interesting ceremony at which the Duke of Cambridge presided. The new theatres are fine structures, complete with modern appliances.

THE New Year honour list contains the names of the following medical men: Dr. Douglas Powell, Physician Extraordinary to the Queen, has been created a baronet; Dr. William R. Kynsey, C.M.G., Medical Officer and Inspector-General of Hospitals in Ceylon, receives the honour of knighthood; and Deputy-Surgeon-General C. B. Moss, C.B., A.M.S., Superintendent Medical Officer of Jamaica, is appointed C.M.G.

THE erection of a new General Hospital at Belfast to commemorate the sixtieth year of the Queen's reign appears to be a very popular proposal. The subscription list has only been open for ten days, and already £34,000 have been collected. The staff of the hospital have contributed £1,300, and one large firm is expected to make a donation of £5,000.

THE Newcastle Infirmary is about to increase its medical staff by the addition of two honorary assistant physicians. The surgeons at the institution have numbered six, whilst hitherto the physicians have been four only. The collections for the new building are proceeding apace, and £80,000 is now promised. The miners of Northumberland and Durham have contributed splendidly.

THE Working Men's Hospital Fund at Leeds appears to desire to follow on the lines of the Birmingham Hospital Saturday Fund, and is about to establish a convalescent home in connection with their fund. The scheme has been drawn out, and will be submitted to the annual general meeting on Jan. 27th. Subscribers only are to be admitted as patients, and it is proposed only to rent a house and furniture in the first instance. We are unaware that such a proceeding has ever been followed before.

THE Christmas number of the *Guy's Hospital Gazette* is a cheery publication, from which medicine, surgery, and the cognate sciences are judiciously eliminated for the season. Besides an admirable portrait of Mr. Cosmo Bonsor, the new treasurer, and a most artistic reproduction by the Autotype Company of the original fresco by Mr. Herbert Draper, which decorates the nurses' dining hall, there are many illustrations of a facetious character, which give life and go to a number which is certainly a credit to the school that has produced it.

THE remains of Louis Pasteur have been laid in their final resting-place in the Cathedral of Notre Dame in Paris. The ceremony was impressive and largely attended by important scientific and other representatives of many countries. The British representatives were the President of the Royal Society, Sir Dyce Duckworth, Sir William Priestley, Professor Evans, of Edinburgh, and Professor Crookshank. The crypt which has been prepared as Pasteur's resting-place is ornamented with mosaic designs, and is surmounted by a cupola in which are figures representing Faith, Hope, Charity, and Science. The inscription above the cupola runs: "Hereux celui qui porte en soi, un Dieu, un idéal de beauté, et qui lui obéit, idéal de l'art, idéal de la science, idéal de la patrie, idéal des vertus de l'evángil."

A SPECIAL meeting of the Council of the Charity Organisation Society was held on January 4th, when Mr. C. H. Warren, secretary of the Metropolitan Provident Medical Association, read a paper on "Provident Dispensaries and their Claim on the Medical Profession." He said that a provident dispensary, to gain general confidence, should have on its committee of management representatives from the benefit members, the medical staff, and the hospital or other institution with which it sought to co-operate; and he laid down the following as conditions which should be observed in the conduct of provident dispensaries: An examination of each applicant for admission, a strict wage limit, the suppression of touting and canvassing for individual medical men, and the representation of the medical profession on the committee or board of management.

